# Specialist training aspirations of junior doctors in Sierra Leone: a qualitative follow-up study

**DOI:** 10.1186/s12909-018-1292-1

**Published:** 2018-08-15

**Authors:** Aniek Woodward, Euphemia Gooding Lake, Natarajan Rajaraman, Andrew Leather

**Affiliations:** 1 King’s Centre for Global Health and Health Partnerships, School of Population Health and Environmental Sciences, Faculty of Life Sciences & Medicine, King’s College London, London, UK; 2 Ministry of Health, Government of Sierra Leone, West African College of Physicians, Freetown, Sierra Leone; 3 King’s Sierra Leone Partnership, King’s Centre for Global Health and Health Partnerships, School of Population Health and Environmental Sciences, Faculty of Life Sciences & Medicine, King’s College London, London, UK

**Keywords:** Junior doctors, Postgraduate medical education, Aspirations, Preferences, Motivations, Sierra Leone

## Abstract

**Background:**

Sierra Leone is pursuing multiple initiatives to establish in-country postgraduate medical education (PGME), as part of national efforts to strengthen the health workforce. This paper explored the career preferences of junior doctors in Sierra Leone; and the potential benefits and challenges with regards to the development of PGME locally.

**Methods:**

Junior doctors (*n* = 15) who had graduated from the only medical school in Sierra Leone were purposively sampled based on maximum variation (e.g. men/women, years of graduation). In-depth interviews were conducted in October 2013, and digital diaries and two follow-up interviews were used to explore their evolving career aspirations until November 2016. Additionally, 16 semi-structured interviews with key informants were held to gather perspectives on the development of PGME locally. Results were thematically analysed.

**Results:**

All junior doctors interviewed intended to pursue PGME with the majority wanting primarily a clinical career. Half were interested in also gaining a public health qualification. Major factors influencing career preferences included: prior exposure, practical (anticipated job content), personal considerations (individual interests), financial provision, and contextual (aspirations to help address certain health needs). Majority of doctors considered West Africa but East and South Africa were also location options for clinical PGME. Several preferred to leave the African continent to pursue PGME. Factors influencing decision-making on location were: financial (scholarships), practical (availability of preferred specialty), reputation (positive and negative), and social (children). Key informants viewed the potential benefits of expanding PGME in Sierra Leone as: cost-effectiveness (compared to overseas specialist training), maintaining service delivery during training years, decreasing loss of doctors (some decide not to return after gaining their specialist degree abroad), and enhancing quality control and academic culture of the local medical school. Major perceived challenges were capacity constraints, especially the dearth of specialists required to achieve training programme accreditation.

**Conclusions:**

This study has provided an insight into the career preferences of junior doctors in Sierra Leone. It is timely as there is increasing political and professional momentum to expand PGME locally. Findings may guide those involved in this PGME expansion in terms of how possibly to influence junior doctors in their career decision-making.

**Electronic supplementary material:**

The online version of this article (10.1186/s12909-018-1292-1) contains supplementary material, which is available to authorized users.

## Background

A prerequisite for universal health coverage and functional health systems is having sufficient numbers of health workers within facilities with appropriate training, skill mix, distribution and performance [1]. The majority of countries experiencing a crisis in human resources for health (HRH) are in Sub-Sahara Africa (SSA), many of them conflict-affected [2]. Conflict-affected countries struggle with a “flight of human capital, mismatches between skills and service needs, breakdown of pre-service training, and lack of human resource data” [3]. The West African Ebola outbreak of 2014–2016 exposed additional fragilities of the affected countries’ health systems [4].

Sierra Leone has a historical shortage of health workers, including medical doctors and specialists [5]. The civil war 1991–2002 [6] and Ebola crisis of 2014–2016 have compounded this situation both directly (a dozen doctors succumbed to the virus [7]), and indirectly by disrupting the production pipeline, accentuating rural-urban maldistribution, and accelerating attrition through emigration. Latest statistics are that Sierra Leone has 0.024 physicians per 1000 population [8], which is far below the minimum WHO recommendation of 2.3 [9] and Ministry of Health and Sanitation (MoHS) estimates of staffing norms to meet the Basic Package of Essential Health Services [10]. The number of specialists in the public sector is 41, compared to the estimated requirement of 144 [10].

Sierra Leone, like many other SSA countries [11], has an established system for undergraduate medical education, but postgraduate medical education (PGME) remains in its infancy.

In-country undergraduate medical education has been offered by Sierra Leone’s only medical school, the College of Medicine and Allied Health Sciences (COMAHS) since 1988. Postgraduate education opportunities, however, have been limited.

The West African Postgraduate Medical College (WAPMC), which includes the West African College of Physicians (WACP) [12] and West African College of Surgeons (WACS) [13], is currently the regional accreditation body responsible for training and recognition of specialists in Sierra Leone. Specialist credentialing by the WAPMC consists of three training milestones: the primary, part 1, and part 2 examinations. The primary examination functions as an entrance examination, “aimed at detecting candidates with good basic medical sciences and pathophysiology competencies” [14]. Successful candidates enter training positions in accredited training institutions as ‘residents’, for a stipulated period (minimum 2 years) before attempting their part 1 examinations, then serve an additional stipulated period (minimum 2 years) before attempting their part 2 examinations. Candidates who pass part 1 are conferred the title of ‘Member’, and part 2 the title ‘Fellow’. WACP and WACS prescribe standards for accredited training programmes, ensure compliance, administer examinations, and run mandatory and supplementary teaching courses. A training programme may be awarded ‘full accreditation’ when it has satisfied minimum training criteria, or ‘partial accreditation’ when it is deemed worthy but falling short of minimum criteria.

**Fig. 1:**
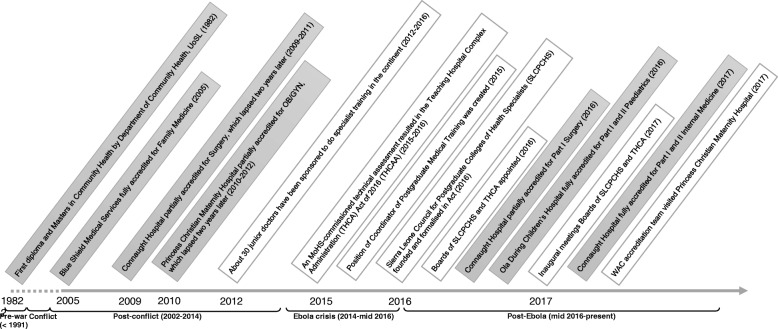
Timeline of key developments of PGME in Sierra Leone (Authors’ compilation)

Increasing PGME is listed as a priority area of cadre production increase in the HRH Strategy 2017–2021, offering the rationale “to develop in-country expertise to mentor and train other clinical cadres, to support research and development of Sierra Leone’s health system and to support growing tertiary care services in the long term” (p69) [15]. Government of Sierra Leone (GoSL) resources for PGME have previously been used for scholarships for specialist training abroad, with major destinations being Ghana and Nigeria. Over 30 doctors have been sponsored under the HRH Strategic Plan 2012–2016 for various programs within the continent [16], of whom a handful returned by early 2017. Changes in budgeting and allocation processes, with greater GoSL discretion, are expected following implementation of the Teaching Hospital Complex Administration (THCA) and Sierra Leone Council for Postgraduate Colleges of Health Specialists (SLCPCHS) Acts. Figure 1 shows a timeline of important developments for establishing local PGME in Sierra Leone.

As in-country opportunities for clinical specialisation are in their formative stages, many doctors take up alternatives abroad, which may contribute to attrition [17, 18]. As Sierra Leone falls under the WAPMC, the ‘West African route’ is the most natural path. Candidates sit the primary exam in Sierra Leone, followed by training in accredited programs, usually in another Anglophone West African country. A popular alternative is the ‘East African route’, but there are instances of study further afield including the UK, Russia, and China. Furthermore, doctors may opt for non-clinical postgraduate studies such as a PhD (approximately 4–6 years), or master’s or diploma in public health, which open doors to more academic and public health careers.

Multiple initiatives are underway to create in-country postgraduate training pathways. These, however, are informed primarily by HRH considerations, with little available data on the prospective trainees’ own views on postgraduate training. A review of published research and grey literature on PGME and specialisation preferences among medical graduates in SSA and countries affected by conflict and the Ebola crisis (see Additional file 1), shows that there is a growing evidence base of career preferences in the region, although no research has focused on junior doctors in Sierra Leone thus far.

This paper aims to explore the career aspirations and specialist training preferences and choices of junior doctors in Sierra Leone. It describes their career aspirations (in the categories of clinical, academic, or public health tracks) and preferences (and where possible actual choices) for specialist training (timing, discipline, and location). In addition, the viewpoints of key informants on potential benefits and challenges of the development of postgraduate medical training in Sierra Leone is described.

## Methods

### Research design

This paper is part of a wider qualitative longitudinal (QL) interview study on the career experiences and aspirations of junior doctors in Sierra Leone.

### Recruitment of participants

Junior doctors were purposively selected based on maximum variation including balance of men/women, years of graduation and career stages (house officer, medical officer, resident), various work/study environments. This type of sampling, which originates from grounded theory, looks for “information-rich” cases [19]. Doctors who graduated from COMAHS from 2002 onwards (i.e. post-civil war) were eligible.

Personal contacts via the King’s Sierra Leone Partnership (KSLP, a collaboration between King’s College London, the Centre for Global Health & Health Partnerships, and institutions in Sierra Leone that helps to strengthen the health system and operates from the country’s main teaching hospital [20]) were accessed to identify potential junior doctors for participation. This was followed by a process of snowballing - asking participants to recommend other participants [21] - generating a list of contact and background details of 48 junior doctors. Those with characteristics required to achieve maximum variation, were invited to participate in the study by the lead researcher (AW) by phone or email. The aim was to achieve a sample size of about 15. This size was chosen to allow for sufficient variation while still being feasible within constraints of the project. In total 28 doctors were approached and 15 agreed to participate. Not answering the phone or non-response via email after initial approach was the main reason for not being recruited.

Key informants were purposively sampled to get a range of perspectives on the benefits and challenges in medical education and workforce development post-conflict (e.g. senior teaching staff at the medical school, policy makers, NGO workers involved with programmes that train health workers, medical students), using a similar recruitment strategy to that used with junior doctors. A list of 26 key informants were identified by AW, 22 were invited and 20 agreed to participate. Two declined, saying they did not have time to be interviewed.

### Methods of data gathering

Five different methods were applied as shown in Table 1, which includes an overview of each method, its objective, approach, and timeline.

**Table 1:** Overview of methods applied for larger qualitative longitudinal study and used for this paper

Method	Objective	Approach	Timeline
1) Semi-structured interviews with key informants	To describe the policy landscape on medical training	A purposively selected sample of key informants (*n* = 20) was interviewed face-to-face. A topic guide^a^ structured interview was used including two group interviews.	Oct ‘13; interviews lasted 27–140 min (63 min average)
2) Document analysis		Relevant policy documentation and statistics were obtained (if available and accessible) via web searches and contacts. These were solely used in the Introduction and Discussion sections of this paper to add context to the results.	Oct ‘13 to Nov ‘16
3) In-depth interviews with junior doctors	To explore career experiences and aspirations	Purposively selected sample of doctors (*n* = 15) was interviewed. Participants were asked to complete a lifeline chart^b^ at the start of the interview to aid reflection on past experiences. Completed charts and a topic guide^c^ helped structure individual interviews. Fourteen were conducted face-to-face and 1 via Skype.	Oct ‘13; interviews lasted 67–126 min (86 min average)
4) Digital diaries	To explore evolving career narratives and aspirations	Participants were asked to record (via email, sms or WhatsApp) accounts of ‘critical events’ related to their career. A guidance sheet was developed to facilitate recordings and emails were sent to invite recordings 4 times per year. 46 digital diaries were collected.	Feb ‘14 to Nov ‘16
5) Follow-up interviews with junior doctors		Follow-up interviews with previously recruited doctors. First interviews focused on experiences related to the Ebola crisis and the second on career aspirations. Eight of 15 junior doctors were initially interviewed via Skype and 7 for the second follow-interviews (6 via Skype; 1 face-to-face).	May ‘15 (interviews lasted 27 min average); Nov ‘16 (interviews lasted 30 min average)

^a^Topic guides for interviews with key informants were based on the literature [17, 46–49] and experiential knowledge. Guides were adapted ^a^ for different types of key informants but generally covered the following topics: evolution of medical school policy; impact of civil war on human resources for health and medical education; coordination, monitoring and regulation of COMAHS; planning and recruitment of medical workforce; quality of medical education; postgraduate medical education and professional development opportunities; financing of medical school; migration/retention of doctors; deployment and distribution of doctors; and gender issues. The interview guide for key informants was piloted with an NGO worker

^b^Lifeline chart recorded key family events (births, deaths, illnesses), places lived, educational and employment history

^c^Topic guide for initial interviews with junior doctors was based on the literature [18, 50, 51] and experiential knowledge and covered the following topics: reasons to start medical education; experiences and financing of medical school; work experiences and options since graduation; attitudes to migration, attitudes to public and private sector work; quality control and regulation; job market and career aspirations; social demands and expectations; and gender issues. A pilot small focus group was conducted with three medical students from Sierra Leone to test the life-line chart and part of the interview guide for junior doctors. No changes to the format of the interview were necessary as students understood all questions

A life-history approach was employed via in-depth interviews with junior doctors. This approach is understood as personal narratives situated in time and context, and was found applicable in a recent publication about career experiences of health workers in post-conflict settings [22, 23].

### Data analysis

All interviews were transcribed and analysed with NVivo 10.2.2. © QSR International (a qualitative computer software programme) by the lead researcher (AW). Thematic analysis was applied to explore relationships and ‘themes’ across the interviews [24]. After familiarisation with the transcripts, data was initially coded and further refined, ordered, reordered, categorised and themed until a final coding framework was identified. This matrix was then applied to all transcripts.

Various recommended approaches [25] were considered to increase validity of the interpreted data. Firstly, through constant comparison, differences and similarities were explored. Differences by gender, cohort year, and preferred area of medical specialism were the main foci. Efforts were made to search for ‘deviant cases’, which are those that might disconfirm emerging themes [19]. Secondly, single counting was used to indicate how strong the evidence of certain experiences was amongst the study cohort [26]. Lastly, initial findings were discussed with co-authors to check if results were aligned with the study objectives.

The results section includes numerous direct accounts, which are particularly important in the life-history approach [27]. The source of each quote cited is indicated as junior doctor (JD), policy maker (PM), senior clinician (SC), or NGO worker (NGO), accompanied by a randomly assigned number for each participant, to illustrate the variety of responses. Unless otherwise specified, interviews were conducted around October 2013. Sources were excluded if it was felt that this could identify participants.

## Results

### Participant characteristics

Table 2 provides an overview of the characteristics of all study participants. Two medical students (group interview) and two policy makers (group interview of four) were excluded as part of the key informant sample, because they did not provide information on the development of postgraduate education. Findings from these key informants will be used in other analyses.

**Table 2:** Study participant characteristics as per November 2013 (unless otherwise specified)

Sample	Characteristic	
Junior doctors (JD) (*n* = 15)	Age	29 years (average); 24–35 years (range)
	Sex	60% male; 40% female
	Graduation year^a^	2015 (*n* = 1), 2013 (*n* = 2), 2012 (*n* = 4), 2011 (*n* = 2), 2010 (*n* = 1), 2009 (*n* = 3), 2008 (*n* = 1), 2006 (*n* = 1)
	Children^a^	yes (33%); no (67%)
	Marital status^a^	single/dating/divorced (60%); married (40%)
	Religion	53% Christian; 47% Muslim
Key informants (*n* = 16)	Sex	75% male; 25% female
	Type	Policy makers (PM) (*n* = 6), Senior clinicians^b^ (SC) (*n* = 6), NGO workers^c^ (NGO) (*n* = 4)

^a^Characteristic as per November 2016

^b^Many of these Sierra Leonean clinicians were also employed by the medical school

^c^Most of these international NGO workers had a clinical background

Four junior doctors (4/15; 3 male, 1 female) were lost during the three-year follow-up study as they did not respond to requests for further interview, although two still provided a diary (one at 8 and another at 12 months follow-up).

### Thematic findings

The analytical framework consisted of five themes: 1) Intentions to pursue PGME; 2) Preferred medical disciplines and careers; 3) Factors influencing career preferences; 4) Preferred locations for PGME and motivations; 5) PGME expansion in Sierra Leone. Table 3 provides an overview of these themes and their sub-themes.

**Table 3:** Overview of themes and sub-themes

Theme	Sub-themes
1. Intentions to pursue PGME	
2. Preferred medical disciplines and careers	2.1 Medical disciplines
	2.2 Clinical career
	2.3 Public health career
	2.4 Academic career
3. Factors influencing career preferences	3.1 Exposure
	3.2 Practical
	3.3 Personal
	3.4 Financial
	3.5 Contextual
4. Preferred locations for PGME and motivations	4.1 Locations
	4.2 Motivations
	4.2.1 Financial
	4.2.2 Practical
	4.2.3 Reputation
	4.2.4 Social
5. PGME expansion in Sierra Leone	5.1 Observed benefits
	5.2 Perceived challenges

#### Theme 1. Intentions to pursue PGME

All (15/15) junior doctors in this study aspired to pursue postgraduate education. Reasons given were having “so much ambition to know things” (JD5), and wanting to progress in their careers:

“Don’t want to get stuck here [in my career] as house officer or medical officer.” (JD13).

“If you do not pursue any other thing, if you do not specialise, if you do not acquire a Masters programme, you just remain a medical officer. Get it? So you only change your status when you pursue something.” (JD8).

Generally, they wanted to start PGME “as soon as possible” after they completed the obligatory internship. Two doctors believed this to be easier:

“I don’t want to relax and then later on try to specialise. It will be difficult” (JD1)

“I think it’s [specialisation] better when you’re young, you have more energy to do this.” (JD15)

Thus, all participants hoped to specialise and soon.

#### Theme 2. Preferred medical disciplines and careers

Junior doctors expressed preferences for a variety of medical disciplines during the study period. Additionally, three types of careers were discussed in interviews, including clinical, public health, and academic.

##### 2.1 Medical disciplines

Table 4 gives an overview of all postgraduate disciplines participants considered during the study period (Oct 2013 to Nov 2016). Eight (6/9 male; 2/6 female) had an ambition to follow a surgical specialty and twelve (6/9 male; 6/6 female) a medical specialty (as defined by the WAPMC).

**Table 4:** Postgraduate specialties junior doctors (*n* = 15) were interested in pursuing during study period (multiple specialties possible per participant)

Postgraduate specialty	Number	Gender
	(%)	M = male; F = female
Public Health^a^	7 (47%)	M = 4; F = 3
Internal medicine^b^	4 (20%)	M = 3; F = 1
Obstetrics & Gynaecology^c^	3 (13%)	M = 2; F = 1
Radiology^c^	3 (13%)	M = 2; F = 1
Family medicine^b^	2 (13%)	F = 2
General surgery^c^	2 (13%)	M = 2
Infectious disease^b^	2 (13%)	M = 1; F = 1
Community Health^b^	1 (7%)	F = 1
Accident & Emergency^d^	1 (7%)	M = 1
Laboratory medicine^b^	1 (7%)	M = 1
Psychiatry^b^	1 (7%)	M = 1
Paediatrics^b^	1 (7%)	F = 1

^a^Includes courses in Public Health, Tropical Health, or International Health. These are typically 1 year Master programmes

^b^Specialty falls under the West African College of Physicians (WACP) [12]

^c^Specialty falls under the West African College of Surgeons (WACS) [13]

^d^This specialty is not recognized in WACP or WACS [12, 13]

Two-thirds (10/15) were set on one specific discipline and a third (5/15) had multiple interests: “I’m still interested in [name specialty] but now I’m also interested in [name other specialty]” (JD4). The majority (10/15) changed their preferences over time (including before and during the study period).

##### 2.2 Clinical career

A clinical career was most popular (13/15) and all of those who hoped to become a clinical specialist were actively pursuing postgraduate education. For example, two residents started training and six others prepared for and/or sat the primary exams during the study period. In addition, everyone hoping to obtain a clinical specialisation seemed knowledgeable about which countries offered their preferred postgraduate education, and medical officers were most likely to actively explore funding opportunities.

“I’m still searching the internet and other places for scholarships and other sponsors.” (JD3).

##### 2.3 Public health career

While half of the sample (7/15) were interested in obtaining a public health related degree, only two doctors intended to enter a public health career. In addition, four hoped for their future career to include some aspect of public health:

“I’d like to do public health as well. But public health can always be done together with [name clinical specialty] later on.” (JD4)

##### 2.4 Academic career

While none of the junior interviewees aspired a full-time academic career, a third (5/15) dreamt of doing some academics ‘on the side’:

“Because my dream is that by the age of forty I should at least have got all the professional accolades I need and then like actually be a full-fledged clinician and doing a bit of academics on the side.” (JD2)

Half (7/15) were involved in teaching medical students either in a formal role as a Demonstrator or Assistant Lecturer within the medical school (5/15) or more informally (2/15) through teaching wards rounds. Only one doctor was pursuing postgraduate course in health professional education. Others (6/15) engaged in teaching, relied on being mentored by senior lecturers:

“I’m an assistant lecturer so I have an immediate boss. She gives me materials, textbooks, sometimes websites, PowerPoint. I have to go over there [to the medical school] and make some modifications and then I do the teaching. Sometimes I go with her to the lecture” (JD8)

Having outlined the specialty and career preferences of junior doctors in this sample, the following section describes the factors underlying such aspirations.

#### Theme 3. Factors influencing career preferences

Five categories of considerations were identified that influence the preferences of junior doctors for type of specialty and career: 1) *Exposure* covers the ways participants are exposed to the different medical disciplines and gained an interest in pursuing these; 2) *Practical* encompasses considerations like perceived content of the specialist job and employability; 3) *Personal* is about their individual interests and beliefs about their abilities; *4) Financial* includes their concerns and successes around funding for postgraduate studies; and 5) *Contextual* considers their aspirations to help address health needs in Sierra Leone and national demands for certain medical specialties.

Table 5 gives an overview of these categories by the types of medical careers (i.e. clinical, public health, academic) and how many doctors mentioned these in their accounts.

**Table 5:** Number of junior doctors (total = 15) who mentioned identified categories of factors in their accounts on three different types of careers (i.e. *clinical, public health, academic)*

	Exposure	Practical	Personal	Financial	Contextual
Clinical	10	8	5	7	6
Public health	5	5	4	6	4
Academic	2	1	4	0	2
Total	17	16	13	13	12

Below, each category is described and examples given of how these influenced the career preferences of participants.

##### 3.1 Exposure

The main reason why junior doctors in this study expressed a medical specialisation preference was through previous clinical exposure. Rotating through various disciplines as a medical student and then intern was mostly commonly mentioned:

“I mean I’ve done rotations in all the disciplines…And I think [specialty] is like the most, it’s more me.” (JD2)

Electives or other international experiences was another way to gain an interest in a specific specialty:

“That [elective in European country] also made me to have more interest in [specialty]. Because when I went there [to that country], things were so different.” (JD11, Oct 14)

An interest in public health was fuelled by the Ebola crisis (2014–2016), with only two doctors intending to get a public health degree before the crisis and six after the crisis:

“I wanted to do [clinical specialty] but in time [due to Ebola crisis] I’ve seen that public health looks at the health issues differently and much more comprehensive. So I’ve gained interest in public health.” (JD15, Oct 16)

Role models were important sources of inspiration during undergraduate and early career experiences. This was particularly evident in gaining a preference for a certain clinical discipline:

“What motivated me to [clinical specialty] is that I mean the [specialist] that we have now, Dr [x] he’s very good. When you’re posted to Dr [last name] the [specialty] he would explain. It was so simple, it was so easy, you would understand everything.” (JD12)

Junior doctors were not only influenced by what senior doctors said and how they taught but also how they perceived their behaviours:

“Almost all [specialists x] in our hospitals here and at COMAHS, they are very nice people, unlike the [specialists y] people. They have some attitude problem. Some of them are like proud.” (JD4)

Observations of what different types of specialists are like, influenced what they wanted to become: “I’m not the [specialist] type of person so I’m not going into [specialty]” (JD8) and “It’s [specialist] more of who I am; It fits into the kind of doctor I think I am” (JD2).

Role models also played a part in getting participants interested in teaching. One doctor had family in academia and another was inspired by house officers when still in medical school:

“When I was in first year, the department of anatomy there were two doctors, Dr [x] and Dr [z]…And they were very instrumental in helping me understand anatomy…I hope I can do as much as they did with me.” (JD6)

##### 3.2 Practical

Junior doctors described several practical factors that influenced their PGME choice. For clinical specialties, participants mainly talked about factors related to the anticipated job content (7/15), location of (1/15) and ease of entry into (1/15) the postgraduate programme.

Phrases used by doctors seeking a surgical career path included: seeing “quick results” (2/15), the opportunity “to work with your hands” (2/15) and “surgeons can act as medic but not the other way around” (1/15). The anticipated amount of standing, however, in surgery deterred two female doctors from pursuing a surgical career. Two other women were drawn towards family medicine because of the anticipated job variety (1/15), the opportunity to “treat the whole family” (1/15), and the opportunity to do it in Sierra Leone, close to their children (1/15). Practical benefits given for internal medicine were being able to “offer your services almost everywhere” (unlike a surgeon who needs a theatre) (1/15), “see results” (1/15), and “because it’s less competitive and I can easily get a placement in that area” (1/15). Factors pushing doctors away from specialising in internal medicine were a dislike for much reading (1/15) and the inability to help some patients (1/15):

“I sometimes think it’s [internal medicine] depressing; seeing patients everyday asking them their complaints. Some patient get better and you feel good. But some patients just get worse every day. And there are some cases there’s nothing you can do about. So it gets depressing.”

The main practical reason for wanting to obtain a public health degree was to become more employable (5/15). Other reasons (multiple possible) were to increase opportunities for entry into research (1/15), to gain promotion (1/15) and to secure a job within the NGO sector (3/15):

“And of course with Ebola I found out there are a lot of opportunities in public health. I mean if you do [public health] there are more opportunities to work for these big [NGO] organisations.” (JD15, Oct 16)

One female doctor wanted to enter a public health career, because it is *less-time consuming* than a clinical career, allowing more time for family:

“[Clinical] specialisation training, not likely. Maybe another Master’s, hopefully sometime in the future. But I have to think of family life as well. And specialisation takes quite a while and you need so much dedication, which I don’t know if I could do right now.” (JD13, Oct 16)

A practical motivation given for teaching medical students is that it *helps preparation for the primary exams*:

“However, I am really enjoying it [teaching]; it gives me an opportunity to get myself well prepared for future post grad exams within a short time.” (JD8)

##### 3.3 Personal

*Individual interest and passion* equally influenced the three types of careers, with four out of 15 doctors mentioning this for each.

“I want to go for something [clinical specialty] that I have passion for.” (JD3)

“I’ve always been interested in [public health].” (JD10)

“In fact I enjoy it [teaching] so much; I have passion for teaching.” (JD5)

The ability to share knowledge and to interact with students led to a passion for teaching. “Helping masses of people” and “looking at health issues differently and more comprehensively” explained an interest in public health. Clinical career interests are fuelled by exposure, practical, and contextual drivers, which are explained under similarly named headers. Another personal driver, however, of clinical specialisation is *perception of own abilities*, which came up in the accounts of two male doctors:

“And it [clinical specialty] suits me because for example physiology and pharmacology they are subjects that I’m well-grounded in so I can easily get into and then it’s my passion you know”

“Because all science students usually say that chemistry is difficult but I find chemistry very easy.”

##### 3.4 Financial

All expressed a concern about payment of postgraduate training. Financial factors were prominent in accounts on decision-making about location of postgraduate training (11/15), clinical training experiences of residents (2/2) (both further described under ‘Preferred location for postgraduate training’) and type of specialty, including public health (6/15) and clinical (7/15).

“Also I have grown to like [clinical specialty] but my worry is that I will not be able to afford to pay for post graduate studies” (JD1, Aug 14)

These worries were based on experiences by themselves and others in being unsuccessful in securing a scholarship.

“I want to get a specialty training so that I can be more helpful. You know, more sharp in doing whatever I want to do to help people. But unfortunately they [Ministry of Health] told me that they are unable to pay for people now.” (JD14)

“Because you have seniors [junior doctors] who have done their primaries…and scholarships are not forthcoming.” (JD5)

A comment by a resident abroad, who did get a scholarship by the government of Sierra Leone, confirms this challenge.

“I was fortunate to go [abroad for specialist education] but most of my colleagues can’t go.” (JD8, Oct 14)

The Ebola crisis further limited availability of such scholarships, at least temporarily:

“I went in for [medical specialty entry] exams set by the West African College and I succeeded in that exam. The success came at a bad time. Because it was then that we had the Ebola outbreak in the country. So all of us who were to go to Ghana or Nigeria or any East African country for postgraduate training, we were summoned to an informal meeting by the Ministry of Health and Sanitation. And so they have to tell us that this is a bad time for the country. It’s so much that we’ll have very small amount of doctors in the country, we cannot afford letting you go, we’re begging you to stay to help the situation.” (JD14, May 15)

A third of junior doctors (5/15) was willing to forsake their first choice should a scholarship become available for another specialty.

“So my [specialty] choice is one; the support is another issue. Because I liked all my rotations. Any rotation I go through I have a special passion for. Because if there’s any time that I can’t afford to go with what I want, I can manage with the other.” (JD9)

Three considered self-funding clinical postgraduate training.

“Because even if it comes to the worst, even if the government cannot provide a grant for me, I will pay for my own.” (JD12)

While two others commented not being able to afford further clinical education without a scholarship.

“I mean it’s not very possible to work and raise up a few monies to pay for yourself for postgraduate studies, if you only work for the Ministry. So for such a situation you expect the Ministry at some point to provide some opportunities [for you] to go and study” (JD15, Oct 16)

None mentioned financial gains as reason for *what* clinical specialty they preferred (although majority did in terms of *where* to specialise; further outlined under ‘Preferred location for postgraduate training’). One person even commented:

“They [colleagues] still ask me “Why can’t you do something else after this?” Because there is no money in [clinical specialty]. Back home you will just be seeing patients for free…I’ve chosen my path. Nothing in my way will make me turn my back.” (JD8, Nov 15)

Funding influenced commencement of a public health related course. Of the six doctors who applied for funding, two secured a scholarship and four were unsuccessful. And of those unsuccessful, two decided to self-fund their studies, while two others could not afford to do so.

“Yes I’m self-funding. Unfortunately, I couldn’t get any scholarships, either national or international, so.” (JD13, Oct 16)

“I wanted to do a Master’s in Public Health online with one of the universities in the UK. I had already applied. And they came back and said the cost was 10,000 dollars. I mean I can’t, there’s no way I’m going to afford that.” (JD2, Nov 2016)

##### 3.5 Contextual

Junior doctors spoke about the health needs of the people in Sierra Leone in decision-making on a certain clinical (3/15) and public health specialty (4/15).

“If there’s anybody who needs healthcare it’s mothers should come first. Because they are like providing the world for us. So I saw [in my internship] the types of pains they [mothers] go through. And I had a change of mind that no I should stay here to help.” (JD14)

“We have a lot of infectious diseases. A lot of tropical diseases to deal with. I find it good, being able to help people through their ailments. But I would like to take that to another level, you know, rather than sitting in the consulting room attending to individual patients, I would like to be able to be in a position to undertake programmes that would be preventative in nature.” (JD6 Oct 16)

Demands of specific clinical specialists was also considered (3/15). One focused on Africa:

“Firstly, in Africa there is a shortage of doctors, especially [clinical specialty doctors]. So I would like to do [clinical specialty] because I intend to work there [in Africa]. And the demand for a [clinical specialty doctor] is high.” (JD4)

A second doctor initially wanted to specialise in a medicine sub-specialty, however, did not see this “forthcoming” in Sierra Leone, where most people cannot afford this sub-specialty treatment and, therefore, changed ambitions towards a specialty in higher demand in the country. A third chose a certain specialty because not many colleagues were willing to:

“Because so many people [junior doctors] want to do [clinical specialty X]. And there’s nobody who wants to do [clinical speciality Y]. And you see your country growing backward. Someone has to take that risk or let me say to specialise in [clinical specialty Y].” (JD11, Oct 14)

In addition, a shortage of lecturers at the medical school was a reason given by two doctors start helping to teach medical students:

“The department is short of lecturers. And I expressed my interest in that area of when I was in medical school. So there was a reason for me to be employed at that department. That’s why. Shortage of lecturers.” (JD8)

#### Theme 4. Preferred locations for PGME and motivations

Having described ‘what’ participants aspire to specialise in and for what reasons, this section outlines ‘where’ they hope to do such postgraduate training and why.

##### 4.1 Locations

Majority of junior doctors in this study considered doing their clinical specialisation in West Africa (12/15), including Sierra Leone, Nigeria and Ghana, but East Africa (5/15), especially Kenya and Uganda, and South Africa (1/15) were also given options. Six considered studying outside of Africa, such as the UK, Germany, Australia and the US. The UK and the Middle East were popular locations for public health related courses.

Several (6/15) expressed a preference for a specific country or region but most (9/15) were open for various options.

“Because I’m now looking for opportunities all over the place…I’m looking for the availability of such courses and looking at the costs and availability of sponsorship and the like.” (JD6, Oct 16)

##### 4.2 Motivations

Four types of motivations can be distinguished: financial (12/15), practical (9/15), reputation (7/15), and social (6/15). Location was less prominent in accounts on the public health career and, therefore, these motivations apply to the *clinical* career only. The most commonly mentioned motivation is described first.

*4.2.1 Financial*

Twelve spoke about finance in relation to location choice of clinical PGME. Many (9/15) described the site of study as being dependent upon *financial opportunities*:

“Wherever there’s an opportunity. Sierra Leone or somewhere else in Africa...Whether there’s a scholarship available.” (JD7)

Location preferences were also affected by *perceptions of costs* involved. Two doctors aspired to study in East Africa because the fees were less expensive than West Africa. Another wanted to go for West Africa because “I think it’s the cheapest for now” and you pay “less than 350 dollars for the [entry] exam” (JD3). The lack of support for fees and living costs when studying abroad made one doctor lean towards staying in Sierra Leone:

“It’s distressing because most our colleagues that are studying are out there, when you communicate with them, they are going through hardship. Imagine studying in another country, it’s hard. It’s hard even to study in your own country. Now you go out of the country: nobody to support you...You have to pay most of your tuition fees, and your food, you have to maybe pay for campus or you rent a hotel.” (JD9)

Experiences of a resident on training abroad confirm this: “I’m not working in [country] so I’m finding it a bit difficult in terms of getting money”. This same person felt the government scholarship - which required a yearly trip to Sierra Leone to request continuation of this funding and renew study leave - was “not enough” to live by. Training in-country also involved financial strength. A resident explained having to self-fund travel to Nigeria and Ghana to complete rotations in sub-disciplines that are unavailable in Sierra Leone.

The prospect of an advantageous salary during and after residency training made one doctor sit the entry exam in a country outside of Africa:

“Once you pass [the primary exam] you enter and have a placement in a certain hospital. You enter a postgraduate programme and in four years time you become a specialist. And in fact, you don’t pay to do the specialty, rather you’re being paid to do the residency. So after four years you become a specialist and I get more money, much more than specialising in Nigeria, and coming back to Sierra Leone” (JD8)

*4.2.2 Practical*

Two practical considerations could be distinguished. The first is *availability of preferred discipline* (8/15). At the start of data collection (Nov 2013), the only specialty available in Sierra Leone was family medicine, which meant that, except those with an interest in becoming a family physician, had to go outside of the country to specialise.

“The problem we have in Sierra Leone is because we don’t have postgraduate training in country so you have to go out.” (JD3)

The second practical consideration is *accessibility of the educational system* (3/15). One junior doctor compared these systems for the US, UK and East Africa:

“For now, I mean America is very difficult to get into the system. All the ones that have gone to sit the USMLE [United States Medical Licensing Exam] are taking a very long year to enter the system. In England there is the PLAB [Professional and Linguistic Assessments Board], if you get the exam right, you still have a long list for you to be entered into the system. So I think now people are going towards East Africa: Kenya, Uganda.” (JD1)

*4.2.3 Reputation*

Doctors in this study judged reputation using online research but also by receiving advise from senior and junior colleagues and via their own international experiences (many travelled and worked within and outside of Africa for electives, conferences, and family visits). This reputation was judged either positively or negatively.

*Positive reputation* (3/15) was especially a ‘pull’ towards higher income countries like Europe, the US and South Africa. One doctor felt the “number one” place to study medicine was [country X] and another felt [country Y] was the best for surgery: “They have Nobel-prize winners in cardiac surgery.”

*Negative reputation* (4/15) on the other hand acted as a push factor outside of West Africa. A medical officer was deterred by the amount of strikes by doctors in this region:

“In Nigeria they are having so many strikes. If you go there for postgraduate training you spent more than the time you are expected to complete. It is frustrating…For Ghana, I think it’s better compared to Nigeria from what I’ve heard about strikes. Generally, in West Africa they have this problem of strikes, strikes, strikes.” (JD1, Oct 16)

Two other junior doctors were hesitant to study in Sierra Leone. One was worried about the sustainability, “We don’t know, when we’re accredited, if it’s going to be a sustainable thing” (JD9), and another did not want to be in the inception stage: “In Sierra Leone when something starts, it’s going to take some time before that thing gets going well.” (JD8). One doctor was in this trial stage and had been enrolled to start surgery specialisation (which got partial accreditation in February 2016). By the end of this study (November 2016), however, this person had not yet started training as welfare issues still had to be resolved. Another resident wished to have trained elsewhere:

“If I had to do this all over again I wouldn’t choose to do this here [in Sierra Leone]. It’s been so difficult. It’s been fraught with so much frustrations and limitations and difficulties that usually I don’t think would exist in other places.”

*4.2.4 Social*

Social ties were a motivation to study in- (3/15) *and* outside (3/15) of Sierra Leone. Two female doctors wanted to be close to their children and therefore would opt to stay.

“Well I think that the best thing to do it [specialisation] is from here [Sierra Leone]. If we’re accredited, then I don’t think I would leave my family behind, especially my daughter, to go to any place to study.” (Oct 16)

A male doctor preferred Sierra Leone as “being away from home, is just, too much.” (Oct 16).

Three others were interested in studying in certain high-income countries partly because they had family and/or friends there.

#### Theme 5. Postgraduate medical education in Sierra Leone

This final results section presents the views of key informants on the potential benefits and challenges of the expansion of PGME in Sierra Leone.

##### 5.1 Observed benefits

Through analysis of the accounts of key informants on postgraduate medical education, four different benefits could be distinguished. First, over a third of informants (6/16) believed that training residents in-country is more “cost-effective” (PM4) than training them abroad, “which is really expensive in so many ways” (SC14). A policy maker added that: “you would be able to train more [residents] with the same resources you train people outside [of Sierra Leone]” (PM8).

A quarter (4/16; of which 2 NGO workers) expressed a second advantage being that residents contribute to “serving your country” (NGO6) whilst in training. Although, as highlighted by another NGO worker, a “hybrid” model might be preferable where residents gain experience *in* Sierra Leone and *outside* where there is “a hospital that actually works and [they can] see what it’s like to go on a functioning ward round” (NGO12).

Third, four others (4/16; of which 2 senior clinicians) felt that having specialist training in-country allows for improved quality control and helps to create an academic atmosphere. Two of these added that such an atmosphere would motivate junior doctors to (more quickly) start specialisation as “there’s something to look forward to” (SC15).

Fourth, several key informants (3/16) explained an underlying reason to develop postgraduate medical education in house is to prevent attrition of doctors.

“But most of the time where we [in Sierra Leone] loose them [junior doctors] is the fact that we do not have the post-graduate training in-country. You know strengthen the accreditation. So somebody [junior doctor] would go for post-graduate [training abroad] and then it’s left with the grace of God to say ‘When I’m through I come back’. Yes but if you train [residents] internally I’m sure we have more percentage we are able to retain.” (PM8)

The issue of doctors going abroad to specialise and not always coming back to their home country - despite being ‘bonded’ to return and work for several years in civil service - was also highlighted by six other informants.

##### 5.2 Perceived challenges

The main obstacle for the development of specialist medical training highlighted by key informants (6/16) were capacity constraints. Participants explained a certain number of human resources, infrastructural requirements, quality control and governance measurements, need to be in place to fulfil the criteria of the WAPMC and for educational Departments to gain temporary, and eventually permanent, accreditation.

“For your institutions to be accredited you have to fulfil certain criteria. The criteria you have to fulfil include number of trainers and the quality of people. The quality is not a problem as I said that’s very high but the number is the problem that we have. And also you need to have certain basic equipment for you to be able to train [residents].” (SC11)

“You [institution] can’t get accredited unless you have a radiology department…you can’t get accredited if don’t have running water in all the taps...Have to have all the sort of political things in place, which technically they’re on the road to that because they [politicians] have written part of the Act of parliament and things. But you [institution] also need the staff to fill it [institution]. And you need the curriculums, the exams, you need the quality control.” (NGO2)

Particularly the dearth of specialists was found problematic. Two NGO workers were concerned about the high costs attached to hiring clinicians from other West African countries to achieve the necessary minimum number of Fellows able to train residents.

An NGO worker highlighted the need to eventually create a “self-sustaining” system (NGO2); where newly trained Sierra Leonean specialists contribute PG training by mentoring residents. This requires junior doctors currently studying abroad to return, which is not easy with the culture of out-migration prevalent amongst the medical workforce: Five out of the six senior clinicians interviewed got their undergraduate and PG medical degrees from foreign Universities. Half of key informants (8/16) highlighted attrition issues amongst doctors.

Positively a handful of local informants (5/16) observed that currently fewer physicians leave the country than several years ago because of increased salaries (SC11,PM3,PM10,PM16) and improved “conditions of service” (SC11,PM16), including availability of “basic equipment in hospitals” (SC11) and “drugs” (PM16), better “prospect for training” (SC7,PM16) and “promotion” (PM4), and generally a more organised health system (PM16). Besides these pull factors, it helped that recently international policies were implemented to reduce “resource snatching from developing countries” (SC7) meaning Sierra Leonean medical graduates “are more or less forced to stay” (SC11).

Another challenge raised by informants concerns the difficulty to attract residents to train in Family Medicine (the only specialty training available in-country at the time of data collection). A senior clinician outlined that although there was capacity to “train as much as eight junior residents” (SC15) in this specialty there was only one in training at the time of the interview. Two NGO workers believed this might be because the value of family physicians is not fully recognised in Sierra Leone. In agreement, a local clinician explained that West Africans previously did not understand the “concept” of family medicine although “it’s catching up now” (SC15).

## Discussion

This study has provided an insight into the career preferences of junior doctors in Sierra Leone. Our sample of junior doctors’ specialty preferences are broadly similar to findings of three comparable studies in Africa [28–30]. A notable difference is the unpopularity of paediatrics. This might be explained by the implementation of Free Healthcare Initiative (FHCI) in 2009, which increased the workload for those working in maternal and child health [31] and likely minimised out-of-pocket payments, making it less financially attractive to work with children.

Female junior participants desired medical over surgery related specialties (as defined by the WAPMC). Comparable results were found in relevant studies from SSA [28, 32] and high-income settings [33, 34]. The anticipated amount of standing involved in surgery was why female doctors in this study were deterred from becoming a surgeon. A similar reason was given by female doctors from Zimbabwe; the nature of surgical work was perceived as too demanding [32]. Other than one female doctor considering time with family as reason to pursue a public health focused career and two preferring to stay in Sierra Leone for PGME to be close to their children, no obvious gender differences were found in this study.

Study findings indicate a variety of factors may influence medical specialty decision-making, which is consistent with results from a European literature review [35]. Factors associated with specialty preferences are more difficult to compare with the literature in SSA, due to differences in terminology and conceptual frameworks. However, two common (and related) themes are the impact of positive experiences during prior undergraduate exposure [29], and personal interest and skills [28].

Results from this research suggest that most junior doctors in Sierra Leone will likely enter a career that has a mix of clinical, academic and public health elements. For example, consultants in Connaught (the main teaching hospital) typically function as clinicians (for public inpatient and outpatient, in addition to private outpatient practice outside of hospital precinct), academics (teaching students from COMAHS, and performing research as local principal investigators) and administrators/public health practitioners (various roles in hospital and the national health system). Possible reasons for occupying multiple roles include the shortage of senior doctors and the attraction of additional income (just for teaching as administrative duties are not reimbursed). Junior doctors may be expected to follow these norms in their own careers.

A significant contribution of the current study, owing to its longitudinal nature, is the finding of fluidity of specialisation preferences over a relative short time span of 3 years, which is consistent with findings from follow-up studies in high income settings [33, 36]. This suggests that preferences may be amenable to being shaped by policy action in desirable directions. Additionally, there may be value in routine longitudinal collection of data on specialty preferences to inform HRH planning.

This study revealed the importance of exposure via clinical rotations and role models for the formation of career preferences. Role modelling has previously been shown to influence medical career choices [37]. A review identified three types of attributes of positive doctor role models: clinical attributes (e.g. knowledge and skills, humanistic behaviours like empathy and compassion), teaching skills (e.g. creating a supportive educational environment), and personal qualities (e.g. effective interpersonal skills, integrity, leadership) [37]. A possible strategy for COMAHS is, therefore, to raise awareness amongst senior clinicians and lecturers of the effects their skills, attitudes and behaviours may have on the career decision-making of their junior colleagues. Additionally, the medical school may consider introducing formal career counselling for medical students and/or interns to guide their specialty choices.

Another finding is the mismatch between junior doctors’ specialisation preferences and HRH planners’ projections of requirements for optimal functioning of the health system (see Table 6) [38]. For instance, Family Medicine, which shows the greatest specialist gap, has failed to attract residents to fill its training capacity, despite being the oldest continually functioning PGME programme in Sierra Leone. The fact that Family Medicine is based in a private instead of a public institution might underlie this problem. Policy action is needed that accounts for both the specialisation preferences of junior doctors in Sierra Leone, and the skill mix needs of the health system. As mentioned above, the introduction of formal career counselling might be beneficial in directing the career choices of young doctors. Another possible strategy is to make more *and* timely scholarships available for these doctors to pursue postgraduate training. This study has shown that financial factors were not just deciding in *what* junior doctors preferred to specialise in, but also *when* they could start PGME and *where* (i.e. country).

**Table 6:** Needs for different medical specialists, adapted from the National Health Sector Strategic Plan 2010–2015 [38]

Specialty	Specialist gap (No. needed)
Family Medicine / General Practice	35
Radiology	29
Paediatrics	28
Internal medicine (Physician Specialists)	21
Obs/gyn	21
Ophthalmology	21
Psychiatry	12
Anaesthesiology	11
Gastroenterology	8
Nephrology	8
Neurology	8
Neurosurgery	8
Otorhinolaryngology (ENT)	7

Our findings suggest that certain factors are favourable for development of PGME locally. Young Sierra Leonean doctors are fully committed to specialising, and prepared to seek this elsewhere if in-country opportunities are not viable. Together with the recent policy developments, the current conditions may represent a special window of opportunity. The costs and challenges to developing PGME can be daunting, as highlighted by key informants of this study, but these investments may be plausibly compensated for by increasing health service capacity and quality in the medium-term, and reducing attrition of doctors in the long run [39–41].

### Limitations

This study has several limitations which should be taken into account. Firstly, results are based on the views of a limited and purposively selected sample of junior doctors and key informants. Consequently, results may not be generalisable to the entire junior doctor population in Sierra Leone. Nevertheless, this exploratory and in-depth study provides a useful basis for further research, which should preferably use a larger randomly selected sample of junior doctors, and possibly have a quantitative or mixed-methods design.

Secondly, the sample of junior doctors (*n* = 15) only included two residents (i.e. doctors in postgraduate training), although it did include one specialising inside and one outside of Sierra Leone. Consequently, results are focused on specialty *preferences* and not actual *choices*. Career preferences change over time, as shown by this follow-up study as in previous research [36, 42–44], and therefore specialty preferences measured at one point in time may be different from eventual career choices. More research is needed on the specialty choices and experiences of Sierra Leonean medical residents, preferably comparing those training in different countries or regions (e.g. Sierra Leone, West Africa, East Africa, US, Europe etc) and those specialising in a variety of medical disciplines.

Thirdly, one fourth of junior doctors (4/15; 3 male, 1 female) dropped out during the three-year follow-up study. Although two still provided a diary (one at 8 months, another at 12 months) these four doctors did not respond to email requests for a further interview. This means possible changes in their specialisation preferences could not be traced. One explanation for drop out is that follow-up interviews were solely conducted via Skype/phone (for financial and safety (Ebola crisis) considerations); online research methods are known for their diminished response rate [45]. Another reason could be that participants did not have the time to continue to be involved in this study. No reasons, however, were given for not responding so their actual reasons might be different from these, meaning it is not possible to provide recommendations to overcome this challenge.

## Conclusion

This study has provided an insight into the career aspirations of junior doctors in Sierra Leone. Additionally, based on viewpoints of key informants, it showed potential benefits (cost-effectiveness, decreasing loss of doctors, enhancing quality control) and challenges (capacity constraints) of the development of PGME in this low-income and crisis-affected country. Study findings are timely - there is currently momentum to expand PGME in Sierra Leone - and may guide those involved in this expansion about how possibly to influence junior doctors in their career decision-making. The qualitative nature and small sample of this study requires caution in generalising findings to other settings. Once PGME has become more established, further research will be needed to explore whether increasing PGME opportunities locally has influenced the career preferences and choices of newly graduated Sierra Leonean doctors, and eventually increased the number of medical specialists in the country.

## Additional file

Additional file 1: “Literature review”. This document contains details (i.e. search strategy and summary results) of the literature review conducted as part of this paper on postgraduate medical education and specialisation aspirations, choices and experiences of junior doctors in Sub-Saharan African and in low-income and /or post-conflict settings globally. (PDF 298 kb)

## Abbreviations

COMAHS: College of Medicine and Allied Health Sciences
GoSL: Government of Sierra Leone
HRH: Human resources for health
JD: Junior doctor
KSLP: King’s Sierra Leone Partnership
MoHS: Ministry of Health and Sanitation
NGO: Non-governmental (NGO) worker
PG: Postgraduate
PGME: Postgraduate medical education
PM: Policy maker
QL: Qualitative longitudinal
SC: Senior clinician
SLCPCHS: Sierra Leone Council for Postgraduate Colleges of Health Specialists
SSA: Sub-Saharan Africa
THCA: Teaching Hospital Complex Administration
TS: Senior teaching staff
WAC: West African College
WACP: West African College of Physicians
WACS: West African College of Surgeons
WAPMC: West African Postgraduate Medical College
