# Supplementary material for: Specialist training aspirations of junior doctors in Sierra Leone: a qualitative follow-up study
Source: BMC Med Educ. 2018 Aug 15;18:199. doi: 10.1186/s12909-018-1292-1 (PMC6094461; doi:10.1186/s12909-018-1292-1)
Supplement: Supplementary file 1 — “Literature review”. This document contains details (i.e. search strategy and summary results) of the literature review conducted as part of this paper on postgraduate medical education and specialisation aspirations, choices and experiences of junior doctors in Sub-Saharan African and in low-income and /or post-conflict settings globally. (PDF 298 kb) [file 12909_2018_1292_MOESM1_ESM.pdf]

Additional File 1: Literature review

Search strategy

Aim

To conduct a literature review on postgraduate medical education and specialisation aspirations, choices and experiences of junior doctors in Sub-Saharan African and in low-income and /or post-conflict settings globally. This review will support the writing of the introduction and discussion of a paper on medical specialisation in Sierra Leone.

Eligibility Criteria:

Population: Medical doctors. Studies including medical students only were excluded.

Settings: Sub-Saharan Africa and countries affected by conflict and the Ebola crisis globally.

Subjects of interest: Postgraduate medical education (PGME) and specialisation aspirations/preferences, choices/decisions and experiences. This includes the nature of the medical career (clinical, academic, public health etc.) and preferences (and where possible actual choices) for specialist training (timing, medical discipline, and location of training).

Study types: All study types, including quantitative and qualitative primary studies but also systematic literature reviews and policy analyses.

Languages: English

Dates: 2002 (post Sierra Leone's civil war) until recent (18 June 2017)

Data sources

Published literature: Systematic search of OVID SP databases MEDLINE, Global Health, and Embase. Search terms are found in Annex A.

Hand search Google Scholar and BMC journals *Conflict & Health*, *Human Resources for Health*, and *Medical Education*. *BMJ Postgraduate Medical Journal. African Journals Online.*

Grey literature: Hand search WHO website and Google. Hand searches used simplified terms like 'medical specialisation' or 'postgraduate medical education' and 'post-conflict' or 'Africa' or 'low-income'.

Results

A scoping review was conducted from June to October 2017, to collate published research and grey literature on PGME and specialisation preferences among medical graduates in SSA and countries affected by conflict and the Ebola crisis globally. A flow-diagram can be found in Annex B of this document. Fourteen sources were eventually included in this review. Three of these (3/14) targeted medical students besides graduates [1–3] and one included also other health workers [4]. Six studies collected data in West Africa [4–9], seven in South-East Africa [1, 2, 10–14], and one in a conflict-affected country in South-Asia [3].

One publication focuses on health workers in Sierra Leone. Wurie et al. 2016 investigated Sierra Leone's rural-urban maldistribution of all health workers, and found that training opportunities are an important factor for satisfaction and decision-making of career trajectories [4].

Numerous publications focus on medical doctors' intentions or decisions to emigrate, and all of them correlate this to postgraduate training opportunities. Three studies surveyed and interviewed doctors in Ghana [5–7], and one in Sri Lanka [3]. All reported that postgraduate training is an important contributor to retention of physicians in-country. Mandeville published various articles on the subject on PG training Malawi [1, 12, 13]. Through discrete choice experiments, she concluded that "indiscriminate expansion of postgraduate training to slow emigration of doctors from SSA may not be effective unless doctors' preferences are taken into account" [13]. A related study from Malawi found that PGME opportunities were the most important factor in participant's career choices [1].

Several surveys describe and analyse covariates of specialty preference patterns among doctors in SSA. In Nigeria, Eze 2011 found the top preferences to be ob-gyn, surgery and paediatrics; and these preferences were influenced by personal interest, career prospects and appraisal of own skills/aptitudes [8]. Ugezu 2012 confirmed these preference patterns, but additionally reported that choices were most influenced by their undergraduate period: having references, good lecturers and a less stressful experience [9]. In Malawi, Muula et al 2002 found top preferences to be public health, internal medicine, and paediatrics [11]. In Uganda, Galukande et al. 2006 report paediatrics and public health the most preferred disciplines. Their analysis centers on reasons for avoiding surgical specialisation, foremost of which are excessive workload, risk of HIV/AIDS, low financial returns and a poor learning environment [10]. In Zimbabwe, Muchemwa 2014's respondents ranked surgery, medicine and anaesthesiology as their top specialties of choice. Their analysis highlights gender differences in preference factors and experience of surgical rotations [2]. Specifically, they find that women prefer non-surgical specialities, because the nature of surgical work is

perceived as too demanding, the work environment and culture being unwelcoming to women, and a lack of female role models.

Mocumbi et al’s report [14] on a MEPI-supported programme to enhance the Internal Medicine residency is instructive in tentatively documenting the broad health system gains resulting from reforms and consequent increased enrolment in a single PGMT programme in a comparable SSA setting.

References

1. Bailey N, Mandeville KL, Rhodes T, Mipando M, Muula AS. Postgraduate career intentions of medical students and recent graduates in Malawi: a qualitative interview study. BMC Med Educ. 2012. doi:10.1186/1472-6920-12-87.

2. Muchemwa FC, Erzingatsian K. Women in Surgery: Factors Deterring Women from Being Surgeons in Zimbabwe. East Cent African J Surg. 2014;19.

3. de Silva N, Samarasekara K, Rodrigo C, Samarakoon L, Fernando S, Rajapakse S. Why do doctors emigrate from Sri Lanka? A survey of medical undergraduates and new graduates. BMC Res Notes. 2014;7:918.

4. Wurie HR, Samai M, Witter S. Retention of health workers in rural Sierra Leone: findings from life histories. Hum Resour Health. 2016;14:3.

5. Clinton Y, Anderson FW, Kwawukume EY. Factors related to retention of postgraduate trainees in obstetrics-gynecology at the Korle-Bu Teaching Hospital in Ghana. Acad Med. 2010;85.

6. Lassey AT, Lassey PD, Boamah M. Career destinations of University of Ghana medical school graduates of various year groups. Ghana Med J. 2013;47:87–91.

7. Amuakwa-Mensah F, Nelson AA. Retention of medical doctors in Ghana through local postgraduate training. J Educ Pract. 2014;5:120–33.

8. Eze BI, Okoye OI, Maduka-Okafor FC, Aguwa EN. Factors influencing choice of medical specialty of preresidency medical graduates in southeastern Nigeria. J Grad Med Educ. 2011. doi:10.4300/JGME-D-10-00101.1.

9. Ugezu A, Modekwe V. House officers’ choice of specialty in Nnewi, South East, Nigeria. AFRIMEDIC J. 2012;3:16–21.

10. Galukande M, Luboga S, Kijjambu SC. Improving recruitment of surgical trainees and training of surgeons in Uganda. East Cent African J Surg. 2006;11:17–24.

11. Muula A, Komolafe O. Specialisation patterns of medical graduates, University of Malawi College of Medicine, Blantyre. Cent Afr J Med. 2002;48:14–7.

12. Mandeville KL, Ulaya G, Lagarde M, Gwesele L, Dzowela T, Hanson K, et al. Early career retention of Malawian medical graduates: a retrospective cohort study. Trop Med Int Heal. 2015;20:106–14.

13. Mandeville KL, Ulaya G, Lagarde M, Muula AS, Dzowela T, Hanson K. The use of specialty training to retain doctors in Malawi: A discrete choice experiment. Soc Sci Med. 2016;169:109–18.

14. Mocumbi AO, Carrilho C, Aronoff-spencer E, Funzamo C, Patel S, Preziosi M, et al. Innovative Strategies for Transforming Internal Medicine Residency Training in Resource-Limited Settings: The Mozambique Experience. Acad Med. 2014;89 8 0:S78–S82.

Annex A: Search terms for bibliographic databases

1. Postgraduate/specialist education terms	2. Medical terms	2. Career preferences/choices terms	4. Setting terms
(postgraduate or post-graduate or specialist) adj2 (education or training or career*)	medical or medical specialist* or doctor* or medical doctor* or medical officer* or house officer* or resident* or registrar* or physician* or general practitioner* or medical school* or medical education or medical training* or medical education/ or medical faculty/	exp Career Choice/ or career* or career path* or career trajectory* or medical pathway* or ((career* or profession* or medic* or rural or private or public) adj (trajector* or path* or pathway* or career* or progress* or course* or choice* or decision* or intention* or preference* or motivation* or incentive* or aspir* or migration or location* or pace or retention or turnover)) or preference* or aspir* or intention*or choice* or decision* or experience*	SubSaharan Africa or Sub-Saharan Africa or Africa* or Africa/ or low-income/ or lowincome/ or developing countr* or ((fragile or unstable or post-conflict or postconflict or conflict-affected or conflict affected or conflict or crisis or war) adj (nation* or state* or country or countries or situation* or context* or setting* or region*))

Annex B: Flow diagram of literature review

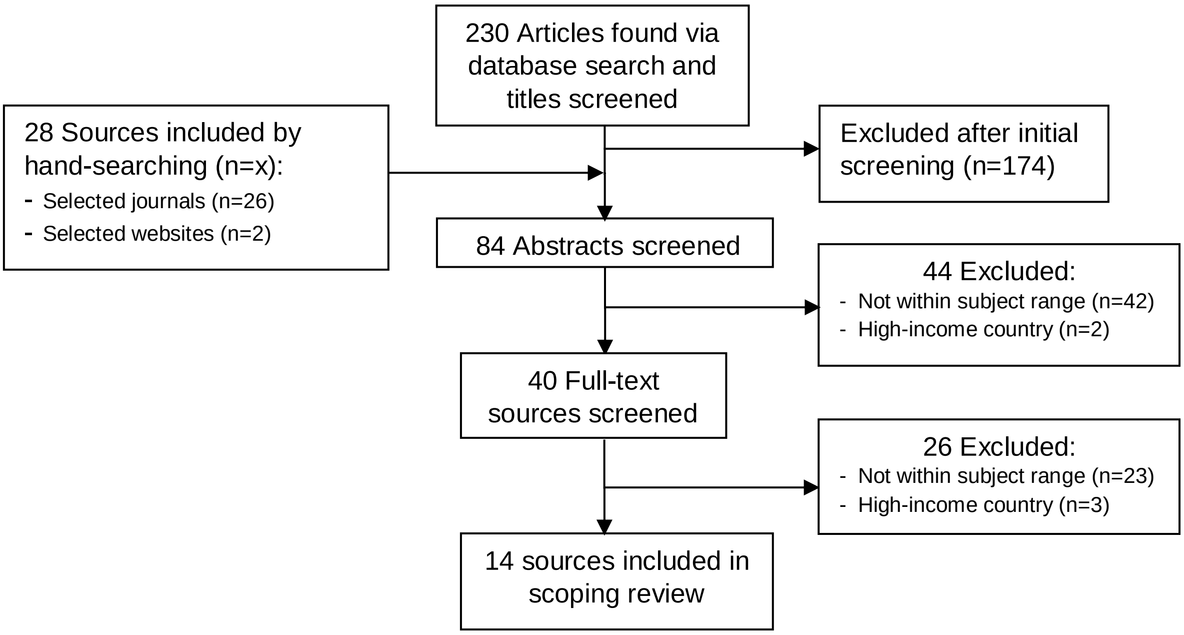

Annex C: Overview of included sources

First Author (Year)	Title of source	Country of data collection	Target population	Covers nature of medical career	Covers details on preferences/ choices for specialist training	If covers preference/choice of medical discipline; type of discipline
Wurie (2016)	Retention of health workers in rural Sierra Leone: findings from life histories	Sierra Leone	Health Workers (doctors, nurses, midwives, CHOs)	No	No	No
Clinton (2010)	Factors Related to Retention of Postgraduate Trainees in Obstetrics-Gynaecology at the Korle-Bu Teaching Hospital in Ghana	Ghana	Ob/Gyn Residents	No	No	Ob/Gyn
Lassey (2013)	Career Destinations of University of Ghana Medical School Graduates of Various Year Groups	Ghana	Doctors	Partial	No	No
Amuakwa-Mensah (2014)	Retention of Medical Doctors in Ghana through Local Postgraduate Training	Ghana	Doctors (enrolled or completed PG training program)	No	Yes	Opth, FamMed, IntMed, Ob/Gyn, PH, Rad, Surg, Uro
Eze (2011)	Factors Influencing Choice of Medical Specialty of Preresidency Medical Graduates in Southeastern Nigeria	Nigeria	Pre-residency Medical Graduates	No	Yes	O&G, Surg, Paeds, IM, CommMed, Rad, Ophth, Path, GP, ENT, Anaes, Psych, Dent
Ugezu (2012)	House Officers' Choice of Specialty in Nnewi, South East, Nigeria	Nigeria	House Officers	No	Yes	Med, Path, Paed, Ob/Gyn, Surg, Anat, Bioch, Physio, Pharm, PH
Galukande (2006)	Improving Recruitment of Surgical Trainees and Training of Surgeons in Uganda	Uganda	Intern doctors, PG surgical trainees, qualified	No	Cursory	Paeds, PH, Surgery

			surgeons, medical administrators			
Muula (2002)	Specialisation patterns of medical graduates, University of Malawi College of Medicine, Blantyre	Malawi	Doctors	No	Yes	PH, IntMed, Paeds
Bailey (2012)	Postgraduate career intentions of medical students and recent graduates in Malawi: a qualitative interview study	Malawi	Medical students and recent graduates	No	Yes	Surg, Anaes, Ob/Gyn, PH, Med subspec, IntMed, Paeds
Mandeville (2015)	Early career retention of Malawian medical graduates: a retrospective cohort study	Malawi	Doctors (graduates 2006-2012)	Partial	No	No
Mandeville (2016)	The use of specialty training to retain doctors in Malawi: A discrete choice experiment	Malawi	Junior doctors	No	Yes	Ophth, PH
Muchemwa (2014)	Women in Surgery: Factors Deterring Women from Being Surgeons in Zimbabwe	Zimbabwe	Doctors and medical students	No	Yes	Surg, Med, Anaes, Ob/Gyn, Onco, PH, Paeds, Path, Ophth, GP, Rad
Mocumbi (2014)	Innovative Strategies for Transforming Internal Medicine Residency Training in Resource-Limited Settings: The Mozambique Experience	Mozambique	N/A	No	No	No
De Silva (2014	Why do doctors emigrate from Sri Lanka? A survey of medical undergraduates and new graduates	Sri Lanka	1st and 4th year medical students, pre-intern medical graduates	No	No	No
